# Preoperative screening and prehabilitation strategies prior to ileocolic resection in patients with Crohn’s disease are not incorporated in routine care

**DOI:** 10.1007/s00384-023-04537-z

**Published:** 2023-10-19

**Authors:** Michiel Thomas Jan Bak, Oddeke van Ruler, Laurents Stassen, Marit Ruiterkamp, Jeanine Hubertina Catharina Arkenbosch, Gerard Dijkstra, Maria Johanna Elisabeth Campmans-Kuijpers, Nico Leonard Ulrich van Meeteren, Bart Chateau Bongers, Mariëlle Romberg-Camps, Sander van der Marel, Frank Hoentjen, Koen Willem van Dongen, Rachel West, Janneke van der Woude, Annemarie Charlotte de Vries, on behalf of the Dutch Initiative Crohn and Colitis (ICC) and Dutch Initiative on Crohn and Colitis – Surgery (ICC-S)

**Affiliations:** 1 Department of Gastroenterology and Hepatology, Erasmus University Medical Center Rotterdam, Doctor Molewaterplein 40, 3015 Rotterdam, GD The Netherlands; 2 Department of Surgery, IJsselland Hospital, Capelle Aan Den IJssel, The Netherlands; 3 Department of Surgery, Erasmus University Medical Center Rotterdam, Rotterdam, The Netherlands; 4 Department of Surgery, Maastricht University Medical Center, Maastricht, The Netherlands; 5 Department of Gastroenterology and Hepatology, University Medical Center Groningen, Groningen, The Netherlands; 6 Department of Anesthesiology, Erasmus University Medical Center Rotterdam, Rotterdam, The Netherlands; 7 Top Sector Life Sciences & Health (Health~Holland), The Hague, The Netherlands; 8 Department of Nutrition and Movement Sciences, School of Nutrition and Translational Research in Metabolism (NUTRIM), Maastricht University, Maastricht, The Netherlands; 9 Department of Epidemiology, Care and Public Health Research Institute (CAPHRI), Maastricht University, Maastricht, The Netherlands; 10 Department of Gastroenterology and Hepatology, Geriatrics and Internal and Intensive Care Medicine (Co-MIK), Zuyderland Medical Center, Sittard-Geleen, The Netherlands; 11 Department of Gastroenterology and Hepatology, Haaglanden Medical Center, The Hague, The Netherlands; 12 Department of Gastroenterology, Radboud University Medical Center, Nijmegen, The Netherlands; 13 Division of Gastroenterology, University of Alberta, Edmonton, Canada; 14 Department of Surgery, Maasziekenhuis Pantein, Boxmeer, The Netherlands; 15 Department of Gastroenterology and Hepatology, Franciscus Gasthuis and Vlietland, Rotterdam, The Netherlands

**Keywords:** Crohn’s disease, Preoperative optimization, Prehabilitation, Ileocolic (re-)resection

## Abstract

**Purpose:**

Recently, recommendations on perioperative care have been published to optimize postoperative outcomes in preoperative patients with inflammatory bowel disease. This study evaluated the current use of preoperative screening and prehabilitation strategies (PS) prior to elective ileocolic resection (ICR) in patients with Crohn’s disease (CD).

**Methods:**

Patients with CD who underwent an elective ICR were identified from a Dutch prospective cohort study. Primary endpoint was to evaluate to what extent IBD-relevant PS were applied in patients with CD prior to ICR according to the current recommendations.

**Results:**

In total, 109 CD patients were included. Screening of nutritional status was performed in 56% of the patients and revealed malnutrition in 46% of these patients. Of the malnourished patients, 46% was referred to a dietitian. Active smoking and alcohol consumption were reported in 20% and 28%; none of these patients were referred for a cessation program. A preoperative anemia was diagnosed in 61%, and ferritin levels were assessed in 26% of these patients. Iron therapy was started in 25% of the patients with an iron deficiency anemia. Exposure to corticosteroids at time of ICR was reported in 29% and weaned off in 3%. Consultation of a dietitian, psychologist, and physiotherapist was reported in 36%, 7%, and 3%. Physical fitness was assessed in none of the patients.

**Conclusion:**

PS are not routinely applied and not individually tailored in the preoperative setting prior to elective ICR in patients with CD. Prior to implementation, future research on the costs and effectiveness of PS on postoperative outcomes and quality of life is necessary.

## Introduction

Approximately 25% of patients undergo intestinal surgery within 10 years after Crohn’s disease (CD) diagnosis [1]. An ileocolic resection and a potential subsequent re-resection of the ileocolic anastomosis are the most common intestinal resections in CD [2].

Intestinal surgery can be perceived as a major life event and has a significant psychological impact on patients with CD. Consequently, anxiety and depressive symptoms can occur in the pre- and postoperative setting [3]. Moreover, overall postoperative complications rates, following ileocolic (re-)resection (ICR) in CD patients, range from 20 to 30%, including severe complications such as intra-abdominal septic complications (IASCs; defined as surgical site infections (SSIs), anastomotic leakage, and/or abscess), extra-intestinal infections, and hemorrhage [4–7]. Critical appraisal of the literature identified nutritional status, physical fitness, CD medication, laboratory parameters, and smoking as (potential) risk factors of postoperative complications in patients with CD [8]. In this line of reasoning, improvement of these several risk factors might reduce the incidence, impact, and/or severity of postoperative complications [8, 9].

Prehabilitation focuses on the preoperative optimization of modifiable factors, concerning the physical and mental condition of the individual, to reduce postoperative complications and to promote an earlier postoperative recovery [10]. Several studies in patients undergoing abdominal surgery for a gastrointestinal malignancy or other indication(s) have shown beneficial effects of individualized preoperative optimization, by improving several domains or physical fitness (i.e., muscle strength and aerobic fitness), resulting in a significant earlier recovery to baseline functional capacity and a significant reduction of postoperative complications [11–13]. These programs may be beneficial in patients with CD [14].

Recently, two multidisciplinary working groups published practical recommendations for perioperative care (preoperative screening and prehabilitation strategies [PS]) for patients with inflammatory bowel disease (IBD) to optimize postoperative outcomes [8, 15]. This study aimed to evaluate the current use of IBD-relevant PS prior to ICR in patients with Crohn’s disease (CD) in current practice according to these practical recommendations [8, 15].

## Materials and methods

### Participants and study design

Between 2017 and 2022, consecutive patients with CD were identified from an ongoing prospective multicenter, national cohort study in eight academic and six non-academic hospitals (Risk Assessment of Postoperative Recurrence in Crohn’s Disease study (RAP-CD study)) [16]. Adult patients (≥ 18 years) scheduled for an ICR for CD were eligible for inclusion. Exclusion criteria comprised of the following: (I) indication for ICR other than CD, (II) absence of preoperative active ileal disease, (III) presence of gastrointestinal malignancy in the resection specimen, or (IV) the presence of a permanent ileostomy. For this retrospective sub-study, consecutive patients were included from three academic and three non-academic hospitals. Patients were excluded in case of emergency surgery and/or if the patient was lost to follow-up within 30 days following ICR.

### Data collection

Baseline and clinical data were retrieved from the medical chart review: date of birth, sex, age at diagnosis, smoking, family history of IBD, (prior) CD medication use, disease phenotype and behavior at time of surgery according to the Montreal classification, previous intestinal resections for CD, and surgical characteristics.

Surgical characteristics comprised indication for surgery, presence of a pre- and/or perioperative intra-abdominal abscesses, surgical approach (laparoscopy or laparotomy), anastomosis or ostomy formation, and type of anastomosis.

Additional data regarding the preoperative assessment, within 2 months to 2 weeks to ICR, were retrospectively collected on (A) nutritional assessment, defined as assessment of preoperative body mass index (BMI) and/or recent unintentional weight loss (defined as > 10% loss of body weight within 6 months to surgery) and malnutrition (defined as recent weight loss, albumin levels < 30 g/L, BMI < 18.5 kg/m^2^, or BMI > 30 kg/m^2^); (B) assessment of physical fitness (muscle strength assessed with hand grip strength or the 30-s chair stand test; cardiorespiratory fitness assessed with a steep-ramp test or other measures) and physical rehabilitation (defined as improvement of physical fitness under supervision, e.g. physical exercise aerobic activity or muscular resistance training); (C) screening and cessation of smoking and alcohol consumption; (D) presence of comorbidity (diabetes mellitus, hypertension, cardiovascular disease(s), renal failure, history of deep venous thrombosis, pulmonary embolism, pulmonary disease, bleeding disorder) and American Society of Anesthesiologists (ASA) score; and (E) preoperative laboratory values (albumin, full blood count [defined as hemoglobin level, hematocrit level, leukocyte, and thrombocyte count], ferritin, C-reactive protein [CRP]). Moreover, preoperative consultations and interventions by psychologist, dietitian, and physiotherapist were recorded. Nutritional interventions included enteral nutritional support (ENS, defined as additional liquid formula to diet, administered orally or by nasogastric tube), exclusive enteral nutrition (EEN, defined as strict liquid monomeric or polymeric formula nutrition, administered orally or by nasogastric tube), parenteral nutritional support (defined as additional liquid formula nutrition, administered via a venous catheter), or total parenteral nutrition (defined as strict parenteral nutrition). If applicable, assessment by a multidisciplinary team (MDT; including at least one gastrointestinal surgeon and one gastroenterologist, often complemented by a gastrointestinal radiologist) was reported to discuss the indication for surgery.

### Outcomes

The primary outcome of this study was the application of IBD-relevant PS in patients with CD prior to an elective ICR in the Dutch routine care, according to the current recommendations for perioperative care in patients with IBD [8, 15]. PS were defined as strategies, performed within 2 months to 2 weeks prior to ICR, assessed and/or performed in routine care to improve the overall health and daily wellbeing of the individual patient on several domains, including nutritional, physical, and psychological status; smoking and alcohol consumption; laboratory parameters; and exposure to CD medication.

These strategies included a nutritional assessment (assessment of preoperative BMI and/or recent unintentional weight loss), a nutritional assessment (and nutritional intervention) by a registered dietitian in case of malnutrition, assessment of physical fitness by a registered physiotherapist or physical therapist, physical rehabilitation (in case of impaired physical fitness), cessation of active smoking and alcohol consumption, preoperative laboratory assessment (within 2 months to surgery) (albumin, full blood count, CRP, ferritin) and treatment in case of abnormal values (e.g., preoperative iron deficiency anemia), treatment of preoperative intra-abdominal abscesses (confirmed by imaging) with antibiotics and/or drainage, cessation/tapering of corticosteroids (defined as prednisolone < 20 mg or equivalent within 6 weeks to surgery), and psychological rehabilitation (defined as one or more consultation(s) with a psychologist).

### Statistical analysis

Descriptive statistical analyses (frequency, percentage, mean, standard deviation (SD), median, and interquartile range (IQR)) were used to describe the research sample. Categorical variables were quoted as the number and percentage. Continuous variables were tested for normality using the Shapiro–Wilk test. Normal distributed variables were presented as mean with a SD, while non-normal distributed variables were presented as median with an IQR. The statistical analysis of data was performed using IBM SPSS Statistics for Windows, version 25.0 (IBM Corp., Armonk, NY, USA).

### Ethical approval

The RAP-CD study was approved by the Medical Ethics Review Committee of the Erasmus Medical Center (METC-2017–482). The study protocol conforms to the ethical guidelines of the 1975 Declaration of Helsinki (6th revision, 2008) as reflected in a priori approval by the institution’s human research committee.

## Results

### Baseline characteristics

During the study period, 133 patients were eligible for inclusion. Twenty-two patients underwent emergency surgery and were therefore excluded. In total, 109 patients were included in the final analysis. Baseline characteristics are displayed in Table 1. The majority of patients were female (69.7%) with a mean age of 38.4 years (SD: 15.1) at time of surgery. Median disease duration at surgery was 6.5 years (IQR: 1.0–13.0). Of the study population, 65.2% of the patients were treated in a tertiary hospital. Mean interval between surgery indication and ICR comprised of 5.1 weeks (SD: 1.8). Most patients (78.0%) had a preoperative ASA score of 2 at time of surgery. Comorbidity was present in 26.6% of the study population.

**Table 1:** Baseline characteristics (*n* = 109)

Female sex, *n* (%)	76 (69.7)
Age at surgery (years), mean (SD)	38.4 (15.1)
Disease duration at surgery (years), median (IQR)	6.5 (1.0–13.0)
Treatment at a tertiary center,* n* (%)	71 (65.2)
Time span of indication for surgery to ICR (in weeks), mean (SD)	5.1 (1.8)
Montreal classification (age), *n* (%)	
* A2: 17–40 years*	66 (60.6)
* A3:* > *40 years*	43 (39.4)
Montreal classification (location of disease), *n* (%)	
* L1: ileal*	65 (59.6)
* L3: ileocolic*	44 (40.4)
* L4:* + *upper gastrointestinal disease*	9 (8.3)
Montreal classification (behavior of disease), *n* (%)	
* B1: non-stricturing, non-penetrating*	22 (20.2)
* B2: stricturing*	67 (61.5)
* B3: penetrating*	20 (18.3)
Previous bowel surgeries for CD, *n* (%)	31 (28.4)
* Segmental colonic resection*	2 (9.7)
* Ileal resection*	3 (6.5)
* Ileocolic resection*	26 (83.9)
Perianal disease at time of surgery, *n* (%)	8 (7.3)
Prior medication exposure, *n* (%)	
* Corticosteroids*	43 (39.4)
* Immunomodulators (thiopurines/methotrexate)*	82 (75.2)
* Biologicals*^a^	74 (67.9)
* Anti-TNF*	74 (100)
* Ustekinumab*	15 (20.3)
* Vedolizumab*	12 (16.2)
ASA classification score, *n* (%)	
* 1*	6 (5.5)
* 2*	85 (78.0)
* 3*	14 (12.8)
* 4*	1 (0. 9)
* Missing*	3 (2.8)
Comorbidity, *n* (%)^a^	29 (26.6)
* Hypertension*	5 (17.2)
* Cardiovascular disease*	8 (27.6)
* Pulmonary disease*	12 (41.4)
* Pulmonary embolism*	2 (6.9)
* Deep venous thrombosis*	2 (6.9)
* Bleeding disorder*	2 (6.9)
Any CD-related medication exposure at time of surgery*, n* (%)^a^	91 (83.5)
* Antibiotic treatment*	11 (10.1)
* Immunomodulators*	33 (30.3)
Corticosteroids^*^	32 (29.4)
* Biologicals, n (%)*	48 (44.0)
* Adalimumab*	21 (43.8)
* Infliximab*	9 (18.8)
* Ustekinumab*	15 (31.2)
* Vedolizumab*	3 (6.2)

*SD* standard deviation, *IQR* interquartile range, *ICR* ileocolic (re-)resection, *CD* Crohn’s disease, *TNF* tumor necrosis factor, *IBD* inflammatory bowel disease, *ASA* American Society of Anesthesiologists physical status

^*^Corticosteroids use is defined as ≥ 20-mg prednisolone or equivalent, 6 weeks prior to surgery

^a^Exposure to one or more biologicals is possible

^b^Presence of multiple comorbidities is possible

Surgical characteristics are shown in Table 2. Most of the patients underwent primary ICR (76.4%), while the remaining patients (23.4%) underwent a re-resection. Primary indication for resection was stenotic disease (58.7%), refractory inflammation/step-up therapy (20.2%), abscess (6.4%), or for other indication (14.7%). Disease localization was restricted to the ileum in 59.6% of patients versus 40.4% of patients who had ileocolic disease at ICR. Preferred surgical approach was laparoscopy (83.5%). A primary anastomosis (93.6%) was created with a preference for a side-to-side (91.2%) and stapled (72.5%) anastomosis. An ostomy was created in 6.4% of the patients.

**Table 2:** Surgical characteristics (*n* = 109)

Type of intestinal resection, *n* (%)	
* Primary ileocolic resection*	83 (76.4)
* Ileocolic re-resection*	26 (23.4)
Primary indication for resection, *n* (%)	
*Refractory inflammation/step-up therapy*	22 (20.2)
* Stenosis*	64 (58.7)
* Abscess*	7 (6.4)
* Other*	16 (14.7)
Disease localization at time of surgery, *n* (%)	
* Ileal*	65 (59.6)
* Ileocolic*	44 (40.4)
Surgical approach, *n* (%)	
* Laparoscopic*	91 (83.5)
* Laparotomy*	18 (16.5)
Primary anastomosis, *n* (%)	102 (93.6)
* Side-to-side*	93 (91.2)
* End-to-end*	3 (2.9)
* End-to-side*	3 (2.9)
* Missing*	3 (2.9)
Creation of an ostomy, *n* (%)	7 (6.4)
Anastomosis techniques, *n* (%)	
* Stapled anastomosis*	74 (72.5)
* Hand-sewn anastomosis*	27 (26.5)
* Missing*	1 (1.0)
* Isoperistaltic anastomosis*	38 (37.3)
* Antiperistaltic anastomosis*	25 (24.5)
* Missing*	39 (38.2)
Surgery duration (in minutes), mean (SD)	127 (47)
* Missing*, *n* (%)	25 (22.9)
Perioperative blood loss (in mL), median (IQR)	50 (0–200)
* Missing*, *n* (%)	61 (49.5)

*SD* standard deviation, *IQR *interquartile range

## Preoperative screening and prehabilitation strategies

### Nutritional status, physical fitness, and psychological status

PS, applied prior to ICR, are displayed in Table 3. All patients were screened and counseled by a gastroenterologist and a surgeon in the preoperative period. More than half of the patients (55.6%) was discussed in an MDT.

**Table 3:** Prehabilitation strategies prior to surgery (*n* = 109)

**Preoperative screening**		**Prehabilitation strategies**	
Assessment in multidisciplinary consultation, *n* (%)	60 (55.6)	-	
Assessment of nutritional status, *n* (%)	61 (55.9)	Dietitian consultation (entire study cohort)	39 (35.8)
		Dietitian consultation in patients assessed for nutritional status, *n* (%)	29 (47.5)
-		Nutritional intervention in patients assessed for nutritional status, *n (%)*	27 (93.1)
		* - Enteral support*	20 (74.1)
		* - Exclusive enteral nutrition*	5 (18.2)
-		* - Total parenteral nutrition*	2 (7.7)
Diagnosis of malnutrition	28 (45.9)	Dietitian consultation for malnourished patients, *n* (%)	13 (46.4)
-		* - Enteral support*	9 (69.2)
		* - Exclusive enteral nutrition*	3 (23.1)
		* - Total parenteral nutrition*	1 (7.7)
Active smoking at time of surgery, *n* (%)	22 (20.2)	Referral to a smoking cessation program, *n (%)*	0
Alcohol consumption, *n* (%)	31 (28.4)	Referral to an alcohol cessation program, *n (%)*	0
-		Psychologist consultation, *n* (%)	8 (6.5)
Assessment of hand-grip strength	0	Physiotherapy consultation, *n* (%)	3 (2.8)
		Physical rehabilitation, *n* (%)	0
Assessment of laboratory values, *n* (%)			
*Albumin level*	26 (31.3)	-	
*CRP*	47 (43.1)	-	
Blood count^*^	52 (47.7)	-	
*Hemoglobin*	70 (64.2)	Assessment of ferritin levels in case of anemia^**^, *n* = 43 (61.4%)	11 (25.6)
		Preoperative iron suppletion in case of iron deficiency anemia^***^ (intravenous or oral), *n* = 8 (61.4%)	2 (25.0)
*Hematocrit*	52 (47.7)	-	
*Leukocyte count*	65 (59.6)	-	
*Thrombocyte count*	66 (60.5)	-	
Diagnosis of a preoperative IASC,* n* (%)	14 (12.8)	Preoperative percutaneous drainage,* n* (%)	2 (14.3)
		Preoperative percutaneous drainage & antibiotics,* n* (%)	4 (28.6)
		Direct surgery,* n* (%)	8 (57.1)
Preoperative corticosteroids use^a^,* n* (%)	32 (29.4)	Tapering of preoperative corticosteroids^b^,* n* (%)	1 (3.1)

*ICR* ileocolic (re-)resection, *SD* standard deviation, *CRP* C-reactive protein level, *IASCs* intra-abdominal abscess

^*^Assessment of blood count includes combination of hemoglobin level, hematocrit level, leukocyte, and thrombocyte count

^**^Anemia was defined as a hemoglobin level < 13.5 g/dL for men and < 12.0 g/dL for women

^**^Iron deficiency anemia was defined as an anemia with a ferritin level of < 100 µg/L

^***^Lowered vitamin B12 was defined as a vitamin B12 level < 148 pmol/L; lowered vitamin D was defined as a vitamin D level of < 30 nmol/L

^a^Corticosteroid use is defined as ≥ 20-mg prednisolone or equivalent, 6 weeks prior to surgery

^b^Defined as < 20-mg prednisolone or equivalent within 6 weeks to surgery

A nutritional assessment was executed in 55.9% of the patients of which 45.9% of the patients were malnourished. In those who had an assessment of the nutritional status, 47.5% of the patients was referred to a dietitian mostly followed by a nutritional intervention (93.1%) which predominantly consisted of ENS (74.1%). Of the malnourished patients, 46.4% was referred to a dietitian. A nutritional intervention was started in all patients with preference for ENS (69.2%). Eight patients (6.5%) and three patients (2.8%) were referred to a psychologist and physiotherapist, respectively. Physical fitness was assessed in none of the patients, and none of the patients were referred for physical rehabilitation.

### Laboratory values and other domains

Preoperative assessment of a full blood count was executed in 47.7% of the patients. Hemoglobin levels were assessed in 64.2%. Preoperative anemia (defined as a hemoglobin level < 13.5 g/dL for men and < 12.0 g/dL for women) was diagnosed in 61.4% of the patients out of whom eleven patients (25.6%) had an assessment of ferritin levels. An iron deficiency anemia was diagnosed in eight of these patients of whom two patients (25%) were treated with oral or intravenous iron suppletion. None of the patients underwent a pre- or perioperative blood transfusion.

A preoperative intra-abdominal abscess was diagnosed in 14.8% of the patients. Six patients underwent preoperative percutaneous drainage of whom four patients were treated with concomitant antibiotics. Direct surgery was the treatment strategy in the other eight patients.

Active smoking and alcohol consumption were reported in 20.2% and 28.4%, respectively. No patients were referred to a smoking and/or alcohol cessation program.

Corticosteroid treatment (prednisolone ≥ 20 mg or equivalent), 6 weeks prior to surgery, was reported in 29.4% and only weaned off in one patient (3.1%).

## Discussion

Recent recommendations have been published for PS in the perioperative care path for patients with IBD undergoing intestinal surgery [8, 15]. Preoperative individualized optimization is recommended for every patient with CD prior to elective intestinal surgery to improve perioperative outcomes. Our cohort study shows that these recommended PS on several domains are not routinely applied and not individually tailored in the majority of patients with CD scheduled for an elective ICR. To improve postoperative outcomes in patients with CD, (cost)-effectiveness data on multimodal PS are required as well as implementation strategies for evidence-based interventions to close this knowledge to care gap.

Recently, the benefits of PS have been proven in patients undergoing (abdominal) surgery for colorectal cancer or hepato-pancreato-biliary surgery (including liver transplantation) for gastrointestinal malignancies or other indications in well-designed (randomized) studies [11–13, 17, 18]. These data may not be directly extrapolated to CD since important pathophysiologic differences exist between patients with IBD and patients with malignancies. These differences mainly constitute the inflammatory and immunocompromised state of patients with CD in combination with other systemic complications (e.g. anemia, malnutrition) [10]. In addition, other important differences include the psychological impact due to the chronic nature of the disease and the often younger age. Therefore, a disease-specific prehabilitation program for patients with CD seems indicated.

This cohort study shows that screening and intervention on key elements of PS are not applied. Even with regard to the elements with sufficient evidence on effectiveness to improve postoperative outcome (i.e. nutrition, cessation of smoking and tapering off corticosteroid use), implementation of these strategies is hampered. Firstly, screening of the nutritional status and the use of nutritional interventions appear largely overlooked in clinical practice since the nutritional status was only assessed in half of the patients in this cohort. Subsequently, nearly half of these patients were malnourished. According to current guidelines, screening for malnutrition is recommended in all preoperative CD patients and consists of at least BMI assessment and/or evaluation of unintentional weight loss [19–21]. A more accurate assessment may consist of screening for alterations in body composition (e.g. sarcopenia) as these alterations are common in patients with IBD and associated with dismal outcomes (i.e. higher surgery risk and postoperative complications) [22]. Several measures, such as imaging techniques and the hand-grip strength, provide the opportunity to screen the body composition [22]. In case of malnutrition, preoperative (enteral) nutritional support and in selected cases EEN have a significant beneficial effect to reduce overall postoperative complications in patients with CD [23, 24]. Furthermore, preoperative EEN has proven to be beneficial in the reduction of CRP levels and the increase of albumin levels. As abnormal values in CRP and albumin are known to be associated with postoperative complications in CD, EEN may be considered in case of abnormalities [8]. To close this knowledge to care gap, referral to a dietitian seems indicated for preoperative IBD patients with signs of malnutrition for an adequate nutritional assessment (including assessment of the body composition) with a potential nutritional intervention.

Secondly, smoking is a well-known risk factor for postoperative complications and postoperative recurrence in patients with CD [19, 20, 25]. In our cohort, active smoking was reported in nearly one out of four patients. None of these patients were referred to a smoking cessation program. A systematic review concluded that cessation of smoking, 6–8 weeks prior to surgery, is cost-effective in the prevention of postoperative complications as compared to standard care in patients who underwent major elective surgery [26]. Although smoking cessation interventions have not been studied specifically in CD, the associations with a detrimental postoperative course are evident. Therefore, the implementation of a smoking cessation program is warranted in clinical preoperative care paths of patients with CD.

Thirdly, a substantial number of patients were exposed to corticosteroids within 6 weeks to surgery and were not tapered in the vast majority. The use of corticosteroids is associated with postoperative infectious complications and is, therefore, discouraged in current guidelines [19, 20]. Regarding the use of anti-TNF agents in the preoperative setting, both guidelines and current literature are conflicting on the association of these agents and the risk of postoperative (infectious) complications [4, 7, 19, 20, 27, 28]. Thus, cessation of anti-TNF agents may be considered, especially in non-responders and/or therapy refractory CD. Evidently, the risk of a flare should be taken into account. Use of vedolizumab and ustekinumab seems to be safe in the preoperative setting [15]. In the preoperative care path, an individualized plan on pre- and/or perioperative CD medication is required to avoid unintentional continuation at surgery.

Optimization of several other features may improve postoperative outcomes in patients with CD. However, literature assessing a potential beneficial effect is virtually absent. In this cohort study, only few patients were referred a psychologist prior to surgery. CD-related surgery is often perceived as last resort of treatment by patients and, subsequently, can result into a negative psychological attitude such as depression [3]. Moreover, patients can experience significant problems leading to limitations in education, work, and physical activities [3]. Thus, screening of the psychological status is important in the preoperative period followed by referral to a psychologist on individual basis [8]. Furthermore, a preoperative anemia is associated with postoperative (infectious) complications in patients with CD [8, 29]. A preoperative anemia was diagnosed in more than half of the patients; however, ferritin levels were only assessed in one out of four patients with an anemia. These low rates may be explained by the fact that laboratory values have been assessed within 2 months to 2 weeks to ICR as optimization, in case of abnormalities, prior to surgery could have been performed. Iron therapy was only started in 25% of the patients with an iron deficiency anemia. In case an anemia is diagnosed prior to IBD surgery, preoperative correction with iron suppletion (preferably intravenous) is indicated [30]. However, the effect on postoperative outcomes is unknown. At last, physical fitness was not assessed in this cohort. Remarkably, an impaired preoperative physical fitness in patients with CD has been observed as compared to patients with colorectal cancer or other colorectal diseases [31]. Preoperative optimization of physical fitness seems to be beneficial for patients with CD to improve postoperative outcomes. Future well-designed studies are necessary to assess the potential effect of optimizing these features in the preoperative period in the IBD population.

The main limitation of this study was the retrospective character of the study. This may have led to missing data on PS due to lack of recording, for instance counseling on cessation of smoking. Since most data on PS can be reliably collected retrospectively, we are convinced on the lack of applied PS prior to an ICR in patients with CD. The relationship of either the presence of risk factors or applying an intervention’s effect on (severe) postoperative complications was not tempted due the potential selection and confusion bias caused by the retrospective study design as it is plausible that the more vulnerable patients have received PS. In addition, we have collected data from six out of fifteen participating centers. None of the participating centers in our study had a standardized preoperative care path/preoperative optimization program for patients with CD at time of data collection. Furthermore, no significant differences in baseline characteristics were observed between patients included in this study versus patients who were treated in the remaining centers. Therefore, we consider the chance for selection and confounding bias to be very limited.

## Conclusion

This cohort study demonstrates that PS are not routinely applied and not individually tailored in the preoperative setting prior to an elective ICR in patients with CD in current routine practice. These findings are not in line with the recommendations of multidisciplinary working groups. Future research on costs and effectiveness of multimodal PS specifically focused on patients with CD on complication rate of surgery and quality of life after intestinal surgery as well as subsequent strategies aiming at broad implementation of PS are required.

## Data Availability

The data underlying this article will be shared on reasonable request to the corresponding author.
